# Influence of Oxygen Partial Pressure during Processing on the Thermoelectric Properties of Aerosol-Deposited CuFeO_2_

**DOI:** 10.3390/ma9040227

**Published:** 2016-03-24

**Authors:** Thomas Stöcker, Jörg Exner, Michael Schubert, Maximilian Streibl, Ralf Moos

**Affiliations:** Department of Functional Materials, Zentrum für Energietechnik (ZET), University of Bayreuth, Bayreuth 95440, Germany

**Keywords:** delafossite, thermoelectric properties, aerosol deposition method (ADM), room temperature impact consolidation (RTIC)

## Abstract

In the field of thermoelectric energy conversion, oxide materials show promising potential due to their good stability in oxidizing environments. Hence, the influence of oxygen partial pressure during synthesis on the thermoelectric properties of Cu-Delafossites at high temperatures was investigated in this study. For these purposes, CuFeO_2_ powders were synthetized using a conventional mixed-oxide technique. X-ray diffraction (XRD) studies were conducted to determine the crystal structures of the delafossites associated with the oxygen content during the synthesis. Out of these powders, films with a thickness of about 25 µm were prepared by the relatively new aerosol-deposition (AD) coating technique. It is based on a room temperature impact consolidation process (RTIC) to deposit dense solid films of ceramic materials on various substrates without using a high-temperature step during the coating process. On these dense CuFeO_2_ films deposited on alumina substrates with electrode structures, the Seebeck coefficient and the electrical conductivity were measured as a function of temperature and oxygen partial pressure. We compared the thermoelectric properties of both standard processed and aerosol deposited CuFeO_2_ up to 900 °C and investigated the influence of oxygen partial pressure on the electrical conductivity, on the Seebeck coefficient and on the high temperature stability of CuFeO_2_. These studies may not only help to improve the thermoelectric material in the high-temperature case, but may also serve as an initial basis to establish a defect chemical model.

## 1. Introduction

With thermoelectric generators, thermal energy can be directly converted into electrical energy. Great efforts have been undertaken in the past few decades to increase the efficiency-characterizing figure of merit (*ZT)*

(1) $$ZT=\frac{S^{2}\sigma }{\kappa }T$$

which depends on the Seebeck coefficient (*S*), electrical conductivity (*σ*), and thermal conductivity (*κ*). If one considers only the electrical parameters, the power factor (*PF*) is an established parameter of thermoelectric materials:

(2) $$PF=S^{2}\sigma$$

*ZT* values above 1 were reported for semiconductors like Bi_2-x_Sb_x_Te_3_ and filled skudderudites like Ba_0.3_Ni_0.05_Co_3.95_Sb_12_ or SnSe [1,2,3,4,5,6,7], and can be further enhanced when optimizing the thermoelectric properties through nanostructuring [8,9,10,11]. However, the commercial application of these materials is limited due to their high synthesis and production costs. In addition, several high-*ZT*-materials contain elements that are not abundant and most materials cannot be processed in an environmentally friendly manner. Stability in oxidizing atmospheres, particularly at elevated temperatures, is another serious issue.

Hence, it is a research trend today to substitute those costly and less abundant thermoelectric materials with inexpensive materials, while sustaining acceptable figures of merit. Whereas thermoelectric materials based on conductive polymers or ceramic-polymer hybrids were investigated for room-temperature applications [12,13,14,15,16,17], oxides are especially attractive at elevated temperatures due to their chemical and high-temperature stability, with limitations to some special oxide classes like doped ZnO [18,19], while not having a negative impact on the environment [20,21,22,23,24]. The key challenge when using oxides as materials in thermoelectric generators is the enhancement of their usually low electrical conductivity. In recent years, considerable good figures of merit for layered p-type cobaltites were reported [25,26]. Even though these materials exhibit good thermoelectric properties, NaCo_2_O_4_ for example is not supposed to be stable against temperature cycling and requires a complex synthesis route due to its highly anisotropic behavior [27].

Concerning n-type thermoelectric oxides, SrTiO_3_ exhibits the best properties, even though it has a low mobility (compared to classical semiconductors); the effective mass is particularly high resulting in a power-factor comparable with Bi_2_Te_3_ at room temperature [28,29]. However, *ZT* is rather low owing to a very high thermal conductivity. Another promising n-type semiconducting oxide is Al-doped ZnO, with a reported *ZT* of 0.24 at 1000 °C, and *ZT* = 0.47 at 975 °C for Al-Ga doped ZnO [24,30]. Keeping this in mind, SrTiO_3_ and ZnO are the only n-type oxide materials reported with reasonably high *ZT* values [31].

In the past, some studies have described copper-iron-oxides and claimed them as promising thermoelectric materials due to their high Seebeck coefficient while sustaining a high electrical conductivity and thermal stability [32,33,34,35]. In this work, we evaluated the thermoelectric performance and the electrical conductivity of the delafossite-type oxide CuFeO_2_, as it depends on the oxygen partial pressure at high temperatures, and found some interesting properties, especially an as yet unknown p-n-transition.

Cu^+^Fe^3+^O_2_ delafossite type oxides belong to the *R*3;^−^*m* space group and have a layered crystal structure. The Cu^+^ ions are coordinated by two O^2−^ ions and form O-Cu-O layers parallel to the *c*-axis, whereas the Fe^3+^ ions are coordinated by six O^2−^ ions in an octahedron [36]. By doping the Fe^3+^ site with divalent 3d cations, the electrical conductivity of the intrinsically p-type CuFeO_2_ can be enormously enhanced [37]. Consequently, doping with tetravalent 3d cations leads to n-type semiconductors [38]. Even though this behavior has been published earlier, the fundamental understanding of the electronic conduction mechanism has still not been fully elucidated.

In this study, the novel aerosol deposition method (often abbreviated as AD method or ADM) is used to obtain dense ceramic thin-films of CuFeO_2_. The AD is based on room temperature impact consolidation (RTIC) of ceramic powders and uses a pressure gradient to accelerate an aerosol of submicron particles through a nozzle to the substrate [39,40]. As the particles impact on the substrate, a dense layer forms by fracture and plastic deformation of the particles on the surface of the substrate [41,42,43,44]. Using this method, thin CuFeO_2_ and dense layers were prepared to study the oxygen partial pressure dependence on the thermoelectric properties, to compare aerosol deposited CuFeO_2_ with conventionally solid-state prepared CuFeO_2_, and to deepen the understanding of their electrical conduction mechanism.

## 2. Experimental

**Figure 1:**
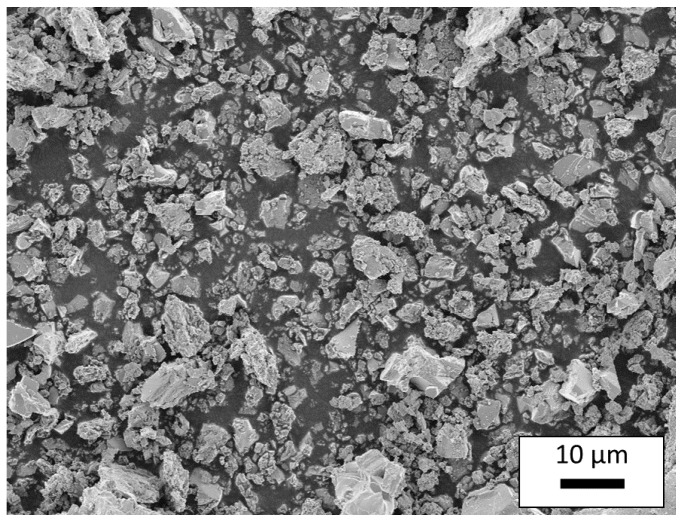
Scanning electron microscope image of a processed starting powder for aerosol deposition.

**Figure 2:**
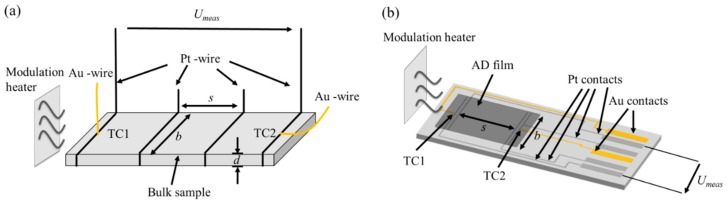
Setup to determine thermoelectric properties of bulk samples (**a**); and aerosol-deposited samples (**b**).

Ceramic CuFeO_2_ powders were synthesized in a conventional mixed-oxide technique using copper(I)-oxide (99.9%, Alfa-Aesar, Karlsruhe, Germany) and iron(III)-oxide (99%, Alfa-Aesar, Karlsruhe, Germany)). These starting materials were processed in a wet planetary ball mill (FRITSCH, Idar-Oberstein, Germany) with cyclohexane as solvent. After milling the powders for 4 h, the solvent was removed in a rotary evaporator (Heidolph Instruments, Schwabach, Germany). To elucidate the influence of the oxygen content of the gas atmosphere during the solid state reaction, CuFeO_2_ fired in 100% N_2_ and CuFeO_2_ fired in 1% O_2_ were synthesized in a high temperature furnace at 1050 °C for 12 h. The obtained delafossite powders were reground in a planetary ball mill using the above mentioned method, sieved with a 90 µm screen in order to reduce agglomerates and finally dried in a furnace at 200 °C for at least 24 h. A scanning electron microscope (SEM, Zeiss, Oberkochen, Germany) image of the calcined and milled delafossite powder used for the AD process is shown in Figure 1. It can be seen that there is a broad particle size distribution ranging from 0.1 to 30 µm which is uncommon for AD processes. Bulk CuFeO_2_ samples were formed into brick shaped pellets, uniaxially cold pressed, and sintered at 1050 °C under the same gas atmosphere as the corresponding starting powder. In order to determine the thermoelectric properties, platinum/gold thermocouples and platinum wires were attached to the sintered samples with platinum conductor paste. Details of the setup are shown in Figure 2.

The AD films were processed in a setup similar to previously published works [45,46,47,48]. It generally contains an aerosol generator, a deposition chamber and a vacuum pump (Edwards Germany, Kirchheim, Germany). In the deposition chamber and in the aerosol generator, a vacuum of 8 mbar is induced. Oxygen serves as a carrier gas at a flow rate of 6 L/min in the aerosol generator where an aerosol is created from the ceramic particles. These particles are transported through a slit-nozzle with an orifice size of 10 × 0.5 mm² and accelerated up to several hundred m/s due to the pressure drop from the aerosol generator into the deposition chamber. The streaming aerosol is ejected on the target at a distance of 3 mm from the nozzle to the substrate and forms dense ceramic layers of several microns. For electrical measurements, AD films were deposited on alumina substrates (CeramTec, Marktredwitz, Germany) of a thickness of 635 µm, a length of 25 mm, and a width of 12.5 mm, on which screen-printed platinum/gold electrodes have been applied before. To obtain XRD patterns, silicon was used as substrate material (CrysTec, Berlin, Germany). The silicon wafers had an orientation of (911), exhibiting no silicon reflexes in the measured XRD diffraction angle range, avoiding substrate influences to the diffraction pattern.

To verify the phase composition of the starting powders and to elucidate the effect of the AD on the crystallography of CuFeO_2_, X-Ray diffraction patterns of both the calcined powder and the aerosol deposited films were taken at room temperature using a PANalytical Xpert Pro system (PANalytical, Almelo, Netherlands) operating with CuK_α_ radiation (1.541874 Å). The intensities were recorded within 2*θ* = 25° .. 60° at a step size of 0.02°. The morphology of the AD films was examined by scanning electron microscopy images of both the cross section and the fracture pattern of the AD samples.

Figure 2 depicts the setup to determine the thermoelectric properties of bulk (a) and aerosol-deposited CuFeO_2_ (b). In both cases, the resistance is measured by a four probe technique with offset compensation (digital multimeter Keithley 2700). By knowing the geometry, the electrical conductivity can be calculated:

(3) $$\sigma =\frac{s}{R\cdot A}=\frac{1}{R}\cdot \frac{s}{b\cdot d}$$

In Equation (3), *s* is the spacing between the inner Pt electrodes, *R* is the measured resistance, *b* the width of the sample, and *d* the thickness of the pellet or the AD film, respectively. The latter was measured by a stylus profilometer (PGK/S2, Mahr, Göttingen, Germany).

To determine the Seebeck coefficient, *S*, an additional modulation heater in front of the samples generated an alternating temperature gradient over the specimens. The temperature difference between the thermocouples TC1 and TC2 was determined via the Au and Pt thermocouple tracks and contact pads, while the thermovoltage *U*_meas_ of the film was measured between the Pt contacts.

Since the Seebeck coefficients of Pt and Au, *S*_Pt_ and *S*_Au_, respectively, are known, the Seebeck coefficient *S* of the delafossite film *versus* Pt can be determined from *U*_meas_. It has to be corrected by the known Seebeck coefficient of platinum, *S*_Pt_. Details of the evaluation of *S* can be found in [49]

(4) $$S=\;S_{Pt}-\frac{U_{meas}}{\Delta T}$$

A periodic voltage, *U*_heater_ = *U*_0_∙cos(2π∙ƒ_mod,heater_∙t)was applied to the modulation heater. It generated the temperature difference Δ*T* = *T*_TC2_ − *T*_TC1_ with the frequency *f*_mod_:

(5) $$\Delta T=\;\Delta T_{0}\cdot \cos\left(2\pi \cdot f_{mod}\cdot t\right)$$

Since heater power and applied modulation heater voltage show a quadratic relation, the temperature difference is modulated with the double frequency as the modulation heater voltage, *i.e.*, *f*_mod_ = 2*f*_mod,heater_ [49]. In Equation (5), Δ*T*_0_ is the amplitude of the temperature modulation, *f*_mod_ stands for the frequency of the temperature modulation, and *t* is the time. *U*_meas_/Δ*T* is determined by a regression analysis of many measured data pairs of the two signals Δ*T*_j_ and *U*_meas,i_. They are plotted according to the following linear equation:

(6) $$U_{\text{meas},j}\;=\;a\cdot \Delta T_{j}\;+\;b$$

The slope, *a,* represents the quotient *U*_meas_/Δ*T* for Equation (6). This method allows elimination of interfering offset voltages. Further details of the data evaluation procedure and accuracies are given in [50]. For our experiments, *f*_mod_ = 12.5 mHz was used, being low enough for our aerosol-deposited specimen to sustain a frequency-independent temperature gradient over the sample [51]. To circumvent interferences between the measurement of the electrical conductivity and the thermopower measurement, a custom-made switching device was used, enabling the automatic alternate measurement of both and electrically insulating them from each other.

In order to determine the influence of the oxygen partial pressure on the thermoelectric properties, the transducers were placed in a tube furnace and gas mixtures of oxygen and nitrogen were applied. The oxygen partial pressure was increased stepwise from 10^−2.6^ bar, being the lower limit of the employed mass-flow-controller, to 1 bar and both the electrical conductivity and the Seebeck coefficient were measured during each *p*O_2_-step while each *p*O_2_ measurement cycle was conducted at 700 °C, 800 °C, and 900 °C.

## 3. Results and Discussion 

### 3.1. Characterization of the Synthesized CuFeO_2_ Powders and AD Films

**Figure 3:**
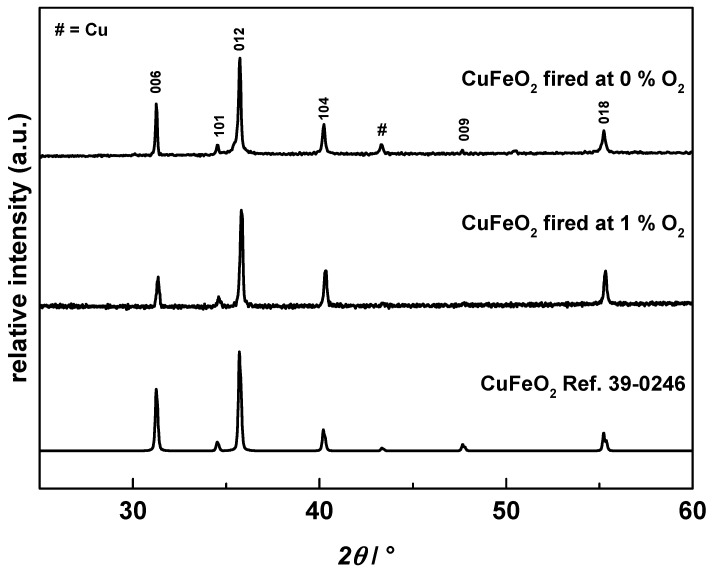
XRD pattern of CuFeO_2_ calcined under pure nitrogen, 1% oxygen mixed in nitrogen and the reference spectrum of CuFeO_2_ JPCD 39-0246).

The crystal structure of CuFeO_2_ was determined by XRD from the calcined powders. Figure 3 shows the pattern of CuFeO_2_ fired in 0% O_2_ (pure N_2_) and the pattern of the 1% O_2_ (1% O_2_, 99% N_2_) fired powder together with the reference pattern (JPCD 39-0246). The characteristic diffraction peaks of CuFeO_2_ can be observed in the pattern, indicating the rhombohedral 3*R* type with the *R*3;^–^*m* space group symmetry [52].

While the XRD pattern of CuFeO_2_ fired in 1% O_2_ appears free from secondary phases, a secondary phase can be seen for the 0% O_2_-fired CuFeO_2_ at *2θ* = 43° (indicated in Figure 3 by #), accounting for elemental copper. We assume this metal impurity is related to the low oxygen content of N_2_ in the alumina tube furnace, resulting in a reduction of Cu_2_O to Cu:

2 Cu_2_O ⬄ 4 Cu + O_2_ ↑ (7)

A similar behavior has already been reported by Zhao *et al.* [53] for delafossites calcined under Ar atmosphere. For further aerosol deposition of powders and for the measurements of the thermoelectric properties, CuFeO_2_ calcined in a mixture of 1% O_2_ in nitrogen was used to avoid traces of the above mentioned copper impurities. The lattice parameters were calculated by Rietveld analyses for Cu-delafossites fired in 1% oxygen to be *a* = 3.0341 Å and *c* = 17.169 Å, which corresponds to pure CuFeO_2_ data reported earlier [54].

**Figure 4:**
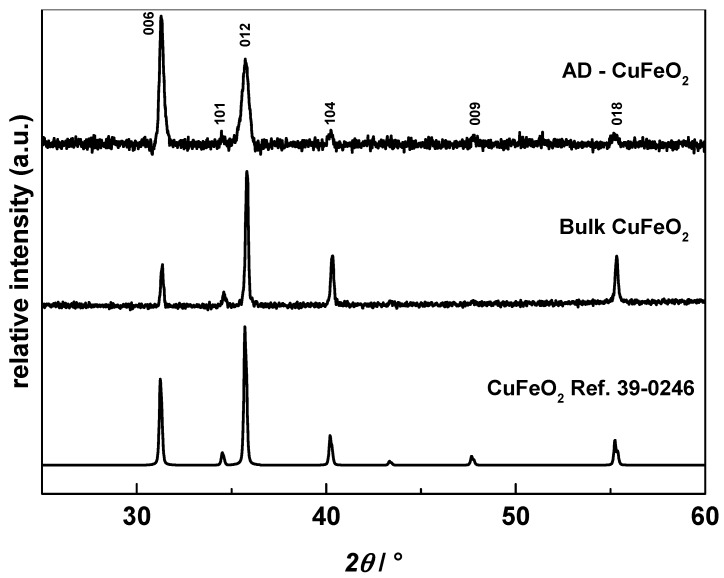
XRD pattern of aerosol-deposited CuFeO_2_ on silicon substrate compared to bulk and reference CuFeO_2_ (JPCD 39-0246).

Figure 4 depicts the XRD patterns of aerosol deposited CuFeO_2_ on silicon substrates. No secondary phases or impurities were observed but the peaks got broader compared to the powder measurements arising from the reduction of the grain sizes during deposition. Based on the Rietveld refinement, the calculated mean grain size of AD CuFeO_2_ was 90 nm compared to 300 nm for the calcined powder. This is a well-known effect in AD films and has been observed for many aerosol-deposited materials [47,55]. In addition, the relative peak intensities differ from the pattern of bulk and reference CuFeO_2_ indicating high lattice strain of aerosol-deposited films, which is also a known phenomenon for aerosol-deposited materials [56,57].

**Figure 5:**
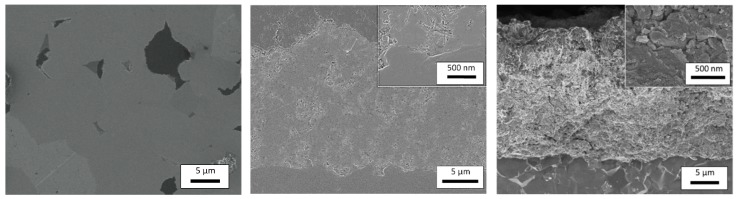
(**a**) Polished cross sectional SEM image of bulk CuFeO_2_; (**b**) Polished cross sectional SEM image of aerosol deposited CuFeO_2_. The inset shows the boundary surface between substrate and film in detail; (**c**) Fractography of aerosol deposited CuFeO_2_ with the inset showing details of the dense CuFeO_2_ film.

SEM cross-sectional images shown in Figure 5a and b indicate crack-free bulk CuFeO_2_ and dense layers of aerosol deposited CuFeO_2_ on alumina, respectively. The film thickness is around 25 µm. From the scanning electron microscope images shown in Figure 5c, the nano-sized microstructure of the aerosol deposited films becomes obvious. The primary particle size ranges from 50 nm to 100 nm, being consistent with the XRD analysis, while agglomerates 400 nm in size are embedded in the nano-sized matrix. This inhomogeneous distribution of grain sizes is due to the particle size distribution of the starting powder for the ADM. While the CuFeO_2_ powders exhibit a *d*_50_ = 6.5 µm (the medium value of the particle size distribution), the *d*_90_ value (90 percent of the distribution lies below this value) of the particles is much larger (*d*_90_ = 16.1 µm). The film forming mechanism for AD layers is supposed to favor mid-range particles around 1 µm, so mainly these particles contribute to the layer formation. The larger particles of the aerosol stream may have less energy to form new ceramic layers and are therefore intercalated between the AD-formed ceramic planes. This phenomenon has also been observed for other aerosol-deposited materials [39,58,59].

### 3.2. Electrical Conductivity of Aerosol-Deposited and Bulk CuFeO_2_

**Figure 6:**
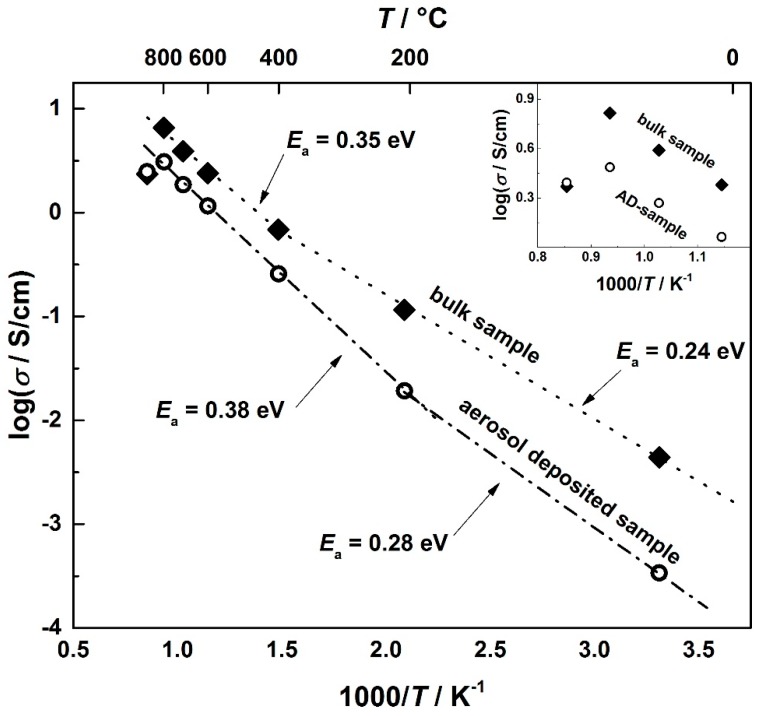
Electrical conductivity of bulk and aerosol deposited CuFeO_2_ and calculated activation energies. The inset displays the abruptly decreasing electrical conductivity at 900 °C in detail.

In order to compare the power factors, *PF* (s. Equation (2)), of AD-processed CuFeO_2_ and standard ceramic-processed delafossites, both the electrical conductivity and the Seebeck coefficient were determined. Figure 6 shows the temperature dependency of the electrical conductivity of AD-CuFeO_2_ and bulk CuFeO_2_ as well as the activation energy of conduction.

AD-processed samples show an offset in the electrical conductivity compared to bulk samples of almost one decade at room temperature, getting smaller with increasing temperature. This effect can be attributed to the microstructure of the deposited CuFeO_2_ films. While sintered bulk samples exhibit almost perfect grain interconnections, AD samples show regions of less densely connected grains. In addition, high strains, as they are common for the room temperature impact consolidation process, impede movements of the charge carriers and diminish the electrical conductivity [47]. With increasing temperature, the grains sinter as well as the microstrain releases, thus enhancing the electrical conductivity, a mechanism observed, e.g., for aerosol deposited MgB_2_ [60].

Since both aerosol-deposited CuFeO_2_ and bulk CuFeO_2_ behave as though thermally activated, the electrical conductivity increases exponentially and can be described by Equation (8); hence, *E*_a_ can be derived from the slope of the Arrhenius-like plot of the electrical conductivity as a function of the inverse temperature.

(8) $$\sigma \;=\;\sigma_{0}\exp\left(\frac{-E_{a}}{k_{B}T}\right)$$

Both aerosol-deposited CuFeO_2_ and bulk CuFeO_2_ indicate a change in the activation energy. While the aerosol-processed sample exhibits a change from *E*_a_ = 0.28 eV to *E*_a_ = 0.38 eV at 200 °C, the bulk sample shows this transition behavior from *E*_a_ = 0.24 eV to *E*_a_ = 0.35 eV at 400 °C. The different transition temperature may be attributed to the microstructure of AD films mentioned above. The values as well as the change of activation energy are consistent with previously published work from Dordor *et al.* [61], where both single-crystals and polycrystalline samples of CuFeO_2_ were investigated.

At temperatures above 800 °C, the electrical conductivity of both samples decreases abruptly, supposedly induced by a certain oxygen loss [37]. To study the origin of this conductivity decrease, the dependency of the electrical transport parameters conductivity (*σ*) and Seebeck coefficient (*S*) on the oxygen partial pressure (*p*O_2_) was investigated at 900 °C for both aerosol deposited and bulk CuFeO_2_.

**Figure 7:**
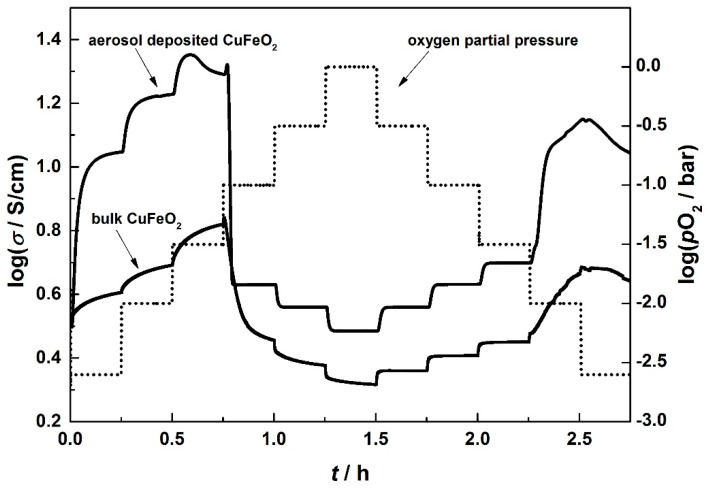
Electrical conductivity of bulk and aerosol deposited CuFeO_2_ with varying oxygen partial pressure at 900 °C. The dotted line represents the oxygen partial pressure.

Figure 7 shows a characteristic measurement cycle. Starting with a pure nitrogen gas atmosphere, the oxygen partial pressure, *p*O_2_, was increased stepwise. Compared to bulk CuFeO_2_, aerosol deposited samples respond much faster to *p*O_2_ steps, promptly reaching an equilibrium state. Below an oxygen partial pressure of 31 mbar (3.1% oxygen), CuFeO_2_ shows a p-type conduction behavior, as can be seen by the increasing conductivity with *p*O_2_. With increasing *p*O_2_, more oxygen is incorporated into the material, resulting in an increased hole concentration, resulting in an increasing electrical conductivity. Thus the *σ* (*p*O_2_) measurement supports the assumption that the abrupt decrease of the electrical conductivity that occurs at 900 °C (displayed in the inset in Figure 6) may be attributed to a loss in oxygen.

**Figure 8:**
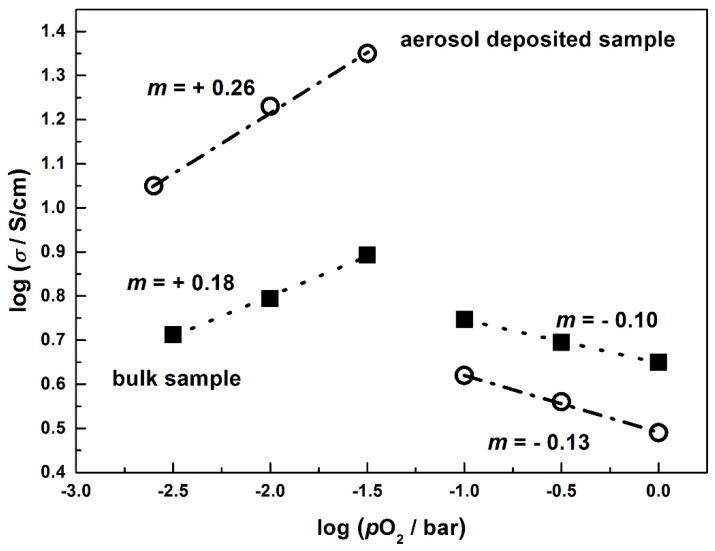
Double logarithmic representation of the electrical conductivity *vs.* oxygen partial pressure at 900 °C for aerosol-deposited CuFeO_2_ and bulk CuFeO_2_.

Astonishingly, the conduction mechanism changes from p-type to n-type behavior at an oxygen partial pressure of 31 mbar, *i.e.*, with increasing *p*O_2_ the electrical conductivity decreases first sharply with a huge conductivity decrease by more than a half decade and then slightly at higher *p*O_2_. This effect is more distinctive for aerosol deposited samples, since the response time for the change in *p*O_2_ is larger compared to bulk samples, not reaching a state of equilibrium. The double-logarithmic representation of the final values in Figure 8 accentuates this.

For typical semiconducting oxides, the electrical conductivity depends on the oxygen partial pressure acc. to Equation (9):

(9) $$\sigma \;=\;\text{const}.\;\cdot pO_{2}{}^{m}$$

In a double-logarithmic plot, the prevalent defect mechanism may be deduced from the slope *m*. While typically slopes of *m* = +1/4 or *m* = −1/6, as they appear for the aerosol-deposited sample, can be explained by classical defect chemical means, see for instance [62,63,64], the slope for the bulk CuFeO_2_ samples can only be explained if one assumes that no equilibration has been settled, *i.e.*, the final values are not equilibrium values.

The abrupt change of the conductivity at around 31 mbar cannot be explained by classical defect chemistry. Instead, we suggest a decomposition of delafossite-type CuFeO_2_ to the corresponding spinel phase CuFe_2_O_4_ and CuO, following Equation (10)

2 CuFeO_2_ + 1/2 O_2_ ➔ CuFe_2_O_4_ + CuO (10)

**Figure 9:**
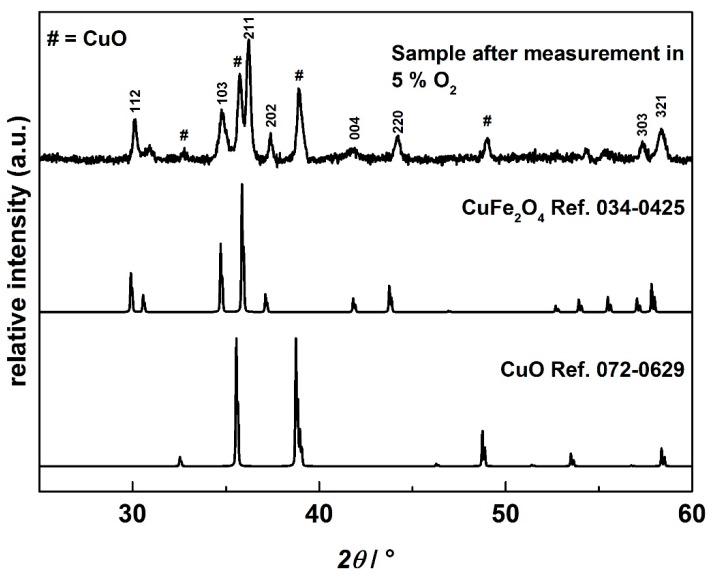
XRD pattern of a sample measured under 5% oxygen for several hours with a reference pattern of CuFe_2_O_4_ (JPCD 34-0425) and CuO (JPCD 39-0629).

According to the Ellingham diagram of CuFeO_2_, this phase change occurs at a *p*O_2_ = 30 mbar at 900 °C [65]. While CuFeO_2_ is a p-type semiconductor, CuFe_2_O_4_ is n-type, being in agreement with our conductivity *vs.* *p*O_2_ data [66]. Such a decomposition reaction could also explain the different distinct conductivity changes between bulk and aerosol deposited films. Since the bulk samples are considerably thicker, oxygen diffusion is by far slower, and a mixed phase consisting of CuFeO_2_ and CuFe_2_O_4_ as well as CuO may be present simultaneously. XRD measurements on samples that have been processed under 5% oxygen also support these assumptions since the XRD pattern clearly showed a mixed phase consisting of both CuFe_2_O_4_ and CuO (Figure 9). No CuFeO_2_ was found since the sample was exposed to the 5% O_2_ atmosphere for a long time (over several hours), so no evidence on the transition phase could be obtained. In order to elucidate this mechanism in particular, measurements of the Seebeck coefficient were conducted.

### 3.3. Thermoelectric Properties of Aerosol Deposited and Bulk CuFeO_2_

**Figure 10:**
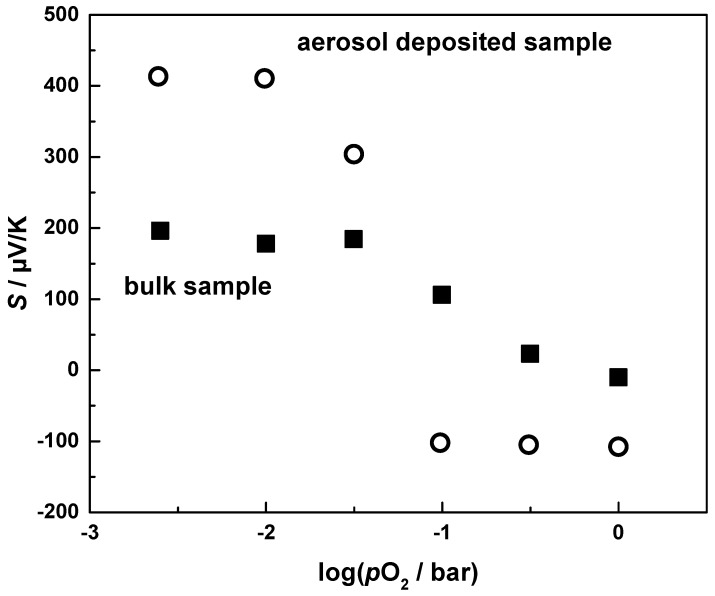
Seebeck coefficient of aerosol-deposited CuFeO_2_ and bulk CuFeO_2_ with varying oxygen partial pressure at 900 °C.

Astonishingly, the Seebeck coefficient of bulk CuFeO_2_ is inferior compared to aerosol deposited CuFeO_2_ at low *p*O_2_. This discrepancy cannot be explained in the manner described for the electrical conductivity, since the thermopower is independent of the geometry (here the interconnection of grains and ceramic layers) and the reduced mobility caused by the high microstrains. Since this behavior is not fully understood, and to elucidate the change of the conduction mechanism from p-type to n-type at *p*O_2_ > 31.6 mbar, detailed measurements of the oxygen dependency of the thermopower were conducted. Figure 10 shows the Seebeck coefficient at 900 °C of both aerosol deposited CuFeO_2_ and bulk CuFeO_2_ as a function of oxygen partial pressure.

With increasing oxygen partial pressure, the Seebeck coefficient of bulk CuFeO_2_ declines slightly up to a *p*O_2_ of 31.6 mbar, thereafter dropping faster with a slope of −115 µV/K per decade *p*O_2_. It becomes even negative at *p*O_2_ = 1 bar (100% O_2_ in the gas), indicating n-type conductivity. Aerosol deposited CuFeO_2_ shows an always constant Seebeck coefficient of +425 µV/K up to an oxygen partial pressure of 31.6 mbar. However, in contrast to bulk CuFeO_2_, the transition from the p-type to n-type conductivity mechanism occurs sharper for aerosol-deposited CuFeO_2_, resulting in a negative Seebeck coefficient of *S* = −100 µV/K at *p*O_2_ = 0.1 bar, which persists at this value up to an oxygen partial pressure of 1 bar.

The changing sign of the Seebeck coefficient supports our assumption of a phase transition of CuFeO_2_ to CuFe_2_O_4_ and CuO with increasing oxygen partial pressure. Since oxygen equilibration kinetics of aerosol deposited CuFeO_2_ samples is much faster compared to bulk CuFeO_2_, the transition appears more pronounced, being completed within one measurement cycle, whereas bulk CuFeO_2_ supposedly exhibits a phase mixture of both CuFeO_2_ (p-type), CuFe_2_O_4_ (n-type) and CuO (p-type), resulting in ambiguous, bipolar thermoelectric effects. With two types of charge carriers present, the Seebeck coefficient of the material is the weighted average of the Seebeck coefficients associated to the different charge carriers as described by Equation (11):

(11) $$S=\frac{\sigma_{n}S_{n}+\sigma_{p}S_{p}}{\sigma_{n}+\sigma_{p}}$$

with the Seebeck coefficients of the materials with different charge carrier types, *S*_n,p_, and their electrical partial conductivities, *σ*_n,p_, respectively [31]. Keeping in mind that the Seebeck coefficients of the n-type and p-type phases have opposite signs, the weighted Seebeck coefficient of a bipolar thermoelectric can be small compared to the purely n-type or p-type conducting materials. The measurements of the Seebeck coefficient of aerosol deposited CuFeO_2_ indicate that at a *p*O_2_ < 31.6 mbar the prevailing phase is CuFeO_2_ with a high thermopower of +425 µV/K. When increasing the *p*O_2_, bipolar effects occur in the transition region, due to the mixture of the decomposing CuFeO_2_ and the emerging CuFe_2_O_4_ and CuO phases. At high *p*O_2_, the transformation ends and the thermoelectric measurements indicate the prevailing n-type CuFe_2_O_4_ phase. For bulk CuFeO_2_, this effect arises much more slowly, resulting in a broader bipolar transition region, and the transformation is not finished at high *p*O_2_ within the measurement cycle, resulting in a bipolar thermopower and a Seebeck coefficient of −15 µV/K compared to −120 µV/K for aerosol-deposited CuFeO_2_ at *p*O_2_ = 1 bar. In fact, it is believed that the bulk sample with a thickness of 500 µm does not reach an equilibrium within half an hour. If one assumes an oxygen kinetic that is diffusion-controlled, one finds equilibration kinetics to be proportional to the square of the thickness of the smallest geometry. In other words, the equilibration kinetics of the AD sample should be faster by a factor of (*d_bulk,sample_/d_AD sample_)^2^ ≈* 20^2^ *≈* 400. Hence, both the thermopower and the conductivity values of the bulk samples appear to be nonequilibrium values and therefore always lie “between” the AD curves. Nevertheless, since the detailed process of aerosol deposition has not yet been fully understood, the consequences of the room temperature impact consolidation on the thermoelectric properties, especially the diverging Seebeck coefficient of bulk and AD processed samples at a *p*O_2_ < 31.6 mbar, remains an open-ended question for further investigations.

**Figure 11:**
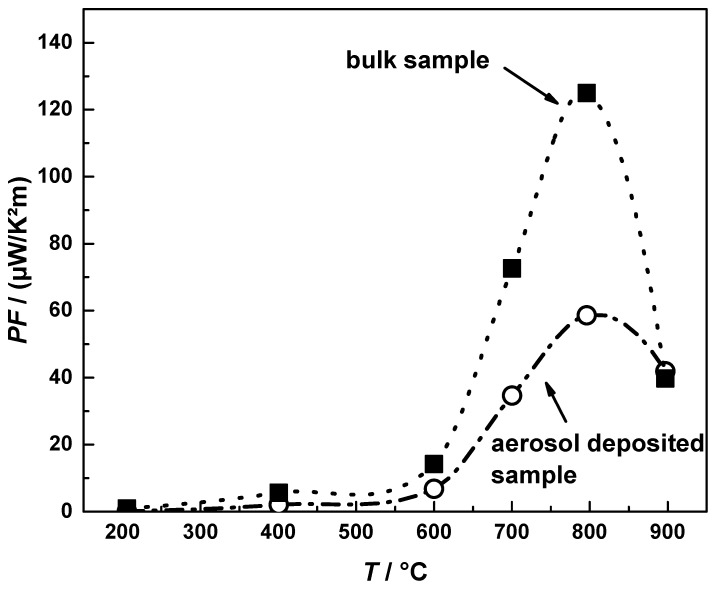
Power factor (*PF*) of aerosol-deposited CuFeO_2_ and bulk CuFeO_2_. The lines are guides for the eye only.

Being of interest as high temperature thermoelectric material, the electrical conductivity and Seebeck coefficients were investigated at temperatures up to 900 °C. Figure 11 shows the power factor (*PF*) of both aerosol deposited CuFeO_2_ and standard processed bulk CuFeO_2_, exhibiting a maximum of *PF* = 59 µW/(K²∙m) at *T* = 800 °C for aerosol deposited CuFeO_2_ and *PF* = 130 µW/(K²∙m) for bulk CuFeO_2_, featuring the same magnitude like other oxide thermoelectrics, e.g., Ca_3_Co_4_O_9_ with *PF* = 225 µW/(K²∙m) or *PF* = 810 µW/(K²∙m) for doped Na_x_CoO_2_ [20].

## 4. Conclusions 

In the present study, the novel aerosol deposition method (ADM) was successfully employed to fabricate dense and crack-free ceramic layers of several microns from the undoped p-type thermoelectric CuFeO_2_ at room temperature with no further heat treatment, thus avoiding interactions with the substrate or the influence of sinter additives. By employing the aerosol deposition method, measurements could be performed on very thin films enabling very fast responses. Since the oxygen partial pressure plays a decisive role during the synthesis and application of Delafossites, XRD studies confirmed that a lowly oxidizing calcination atmosphere is essential for the preparation of single phase CuFeO_2_. The process window, however, is small since at higher oxygen partial pressures, *p*O_2_ > 30 mbar at 900 °C, a phase transition from CuFeO_2_ to the spinel-type CuFe_2_O_4_ and CuO occurs.

Astonishingly, we observed a sudden change of conduction from p-type to n-type at an oxygen partial pressure of *p*O_2_ = 30 mbar. While the electronic structure of CuFeO_2_ can be calculated by an enhanced local spin density approximation [67], this change in the conduction mechanism at a defined oxygen partial pressure has not been observed yet. Investigations on changing valance states of the copper and iron sites in CuFeO_2_ were also conducted in order to establish a defect chemical model [68]. However, we propose that the change is based, for instance, (at least partly) upon a phase transition from p-type semiconducting CuFeO_2_ to n-type CuFe_2_O_4_ and CuO, resulting in a bipolar thermoelectric material. While the thermoelectric properties of the n-type phase are inferior to the p-type CuFeO_2_, this material system can be of interest for use in thermoelectric generators, since both p-type and n-type materials can be precisely tailored only by defined process conditions based on the identical starting thermoelectric material. Nevertheless, detailed defect chemical investigations, particularly more measurements of electric transport parameters combined with other non-electrical analytical means, need to be conducted at defined and especially low-oxygen partial pressures in order to develop a comprehensive defect model of CuFeO_2_. The measurements shown in this study may serve as an initial basis. Furthermore, the influence of dopants needs to be studied to tailor the thermoelectric properties, and detailed measurements on the thermal conductivity of thin aerosol-deposited films deserve further investigation since the reduction in grain size, resulting from the room temperature impact consolidation effect, could lead to a reduction of the thermal conductivity of CuFeO_2_, probably due to increasing phonon scattering at grain boundaries thereby increasing the thermoelectric performance of delafossites.
